# Yeast Strains as Probiotic and Postbiotic Agents for the Agglutination of Enteric Pathogens: A Preventive Approach

**DOI:** 10.3390/pathogens14020113

**Published:** 2025-01-24

**Authors:** Michelle Cerdán-Alduán, Josune Salvador-Erro, Ana Villegas-Remírez, David García-Yoldi, Ana Ceniceros, Yadira Pastor, Carlos Gamazo

**Affiliations:** 1 Department of Microbiology and Parasitology, Navarra Medical Research Institute (IdiSNA), University of Navarra, 31008 Pamplona, Spain; mcerdan.3@alumni.unav.es (M.C.-A.); jsalvador.1@alumni.unav.es (J.S.-E.);avillegasre@alumni.unav.es (A.V.-R.); ypastor@unav.es (Y.P.); 2 LEV2050, Polígono Industrial Plazaola, Manzana E, Nave 10, 31195 Aizoain, Spain; dgarcia@lev2050.com (D.G.-Y.); acmedrano@alumni.unav.es (A.C.)

**Keywords:** yeast, postbiotic, probiotic, agglutination, adhesion, gastrointestinal infection

## Abstract

This study evaluates the potential of various yeast strains as probiotic and postbiotic agents for agglutinating enteric pathogens, offering a preventive approach to gastrointestinal infections. Different yeast species were tested in vitro against a range of pathogenic bacteria, including enterotoxigenic *Escherichia coli* ETEC, *Shigella flexneri*, *Salmonella enterica* serovar Typhimurium, and *Salmonella enterica* serovar Enteritidis, to assess their capacity for pathogen agglutination. Additionally, inactivated yeasts were obtained using a novel chemical treatment and employed to explore their efficacy as postbiotic agents. The results suggest that both live and inactivated yeasts are able to agglutinate the different pathogens, potentially limiting bacterial colonization. Notably, we also demonstrated that *Wickerhamomyces anomalus*, *Saccharomyces cerevisiae*, and *Pichia fermentans*, exhibiting agglutination activity, were capable of reducing bacterial adhesion to HeLa cells in vitro. This research highlights yeast’s probiotic and postbiotic potential and supports the development of novel yeast-based products for preventing enteric infections.

## 1. Introduction

Probiotics are becoming increasingly popular in the healthcare industry, and by 2024, supplement sales are expected to reach USD 35–65 billion [1]. According to the current definition, probiotics are live microorganisms that, when administered in adequate amounts, confer a health benefit for the host [2]. Probiotics act by restoring the equilibrium within the gut microbiota, establishing beneficial bacterial colonies, and interacting with immune cells within the gut-associated lymphoid tissue. These actions collectively enhance processes such as digestion, skin health, and immune system function and can also influence mental health via the modulation of the gut–brain axis [3]. However, there is a rising concern regarding the capability of bacterial probiotics to transfer resistance genes to pathogenic bacteria [4], which, along with the safety restrictions for immune compromised patients, has prompted the search for alternatives [5].

Yeast probiotics have been less studied; however, recent research centered on generally recognized as safe (GRAS) yeasts has highlighted their innate ability to influence physiology, metabolism, and immune homeostasis in the colon. As a result, yeasts have emerged as a promising probiotic source, offering numerous advantages similar to those of traditional bacterial probiotics [6].

Particularly, it has been reported that some live yeasts, such as *Saccharomyces cerevisiae* var*. boulardii,* improve the resistance of the intestinal ecosystem to infectious pathogens such as *Clostridium difficile*, *Escherichia coli*, *Shigella flexneri*, *Vibrio cholerae*, *Candida albicans*, or *Salmonella enterica* serovar Typhimurium [7]. The mechanisms involved encompass immune activation as well as the prevention of colonization through competitive exclusion or the secretion of antimicrobial substances [8].

In recent years, postbiotics, which consist of non-viable microorganisms and their components, have gained significant attention as an alternative to probiotics, due to their stability, safety, and beneficial health effects [2,9,10,11]. Interestingly, some yeast postbiotics have been reported to have immunomodulatory properties due to specific cell wall components. These structures are detected as microbe-associated molecular patterns (MAMPs) with potent immunomodulatory effects on innate immunity by inducing a trained, innate immune response [12]. Among these components, β-glucans have been widely studied for their ability to bind to pattern recognition receptors (PRRs), such as Dectin-1 and complement receptor 3 (CR3), activating macrophages, NK-cells, and consequently T- and B-cells [13]. Mannoproteins are exposed in the external layer of the cell wall and are therefore the most accessible MAMPs for the PRRs. Though mannoproteins have been less investigated than β-glucans, their role in host immune response has been demonstrated [14].

Given the high morbidity and mortality associated with enteric pathogens [15], exacerbated by the growing challenge of antibiotic resistance [16], this study focuses on the potential of yeast probiotics and postbiotics against significant bacterial enteropathogens. To obtains postbiotics, we used for the first time the intercalating agent binary ethylenimine (BEI) in combination with formaldehyde (BEI/FA), which has been previously described for bacterial inactivation [17]. Subsequently, we analyzed the agglutination capacity of yeast products on selected enteric pathogens, such as enterotoxigenic *Escherichia coli* (ETEC), *Salmonella enterica* serovar Typhimurium*, Salmonella enterica* serovar Enteritidis, and *Shigella flexneri,* to reduce their infective load as a preventive strategy against enteric infections. Thus, we further investigated their impact on bacterial adherence and invasion of epithelial cells in vitro. The results revealed that the incubation with either live or inactivated yeasts triggered the coagglutination of ETEC, *Salmonella enterica* serovar Typhimurium, and *Salmonella enterica* serovar Enteritidi*,* but not *S. flexneri.* Moreover, due to the use of various sugars as inhibitors, our results suggest that ETEC agglutination may be mediated through the binding of yeast mannoproteins to the ETEC type 1 fimbriae. Remarkably, this agglutination led to a reduction in ETEC infection in vitro, highlighting the potential of yeast probiotics as a protective strategy against enteric infections.

## 2. Materials and Methods

### 2.1. Strains and Growth Conditions

In this study, nine different yeast species were employed: *Metschnikowia pulcherrima, Papiliotrema terrestris, Pichia fermentans, Rhodotorula glutinis, Rhodotorula mucilaginosa, Saccharomyces cerevisiae, Schizosaccharomyces pombe, Torulaspora delbrueckii*, and *Wickerhamomyces anomalus*. All the strains were kindly provided by the company LEV2050, an industrial microbiology company specializing in the isolation, characterization, and industrial production of microorganisms (https://www.lev2050.com/, Pamplona, Spain). The strains were isolated from agri-food resources and are the property of the company. As such, they are deposited in the LEV2050 cellbank and are not publicly available. These strains were stored in Cryobeads (Microkit laboratories, Madrid, Spain) at −80 °C until use. Yeasts were grown in YPD medium containing 1% yeast extract (Condalab, Madrid, Spain), 2% peptone (Condalab, Madrid, Spain), and 2% dextrose (Panreac-BioChemica, Barcelona, Spain) for 48 h under constant agitation conditions (180 rpm) at 28 °C. YPD agar plates containing 1.5% agar were also used where indicated.

The bacterial strains employed in this study were the following: Enterotoxigenic *Escherichia coli* H10407 (ATCC 35401), *Salmonella enterica* serovar Enteritidis (ATCC 25928), *Salmonella enterica* serovar Typhimurium (ATCC 43971), *Shigella flexneri* 2a, and *Pseudomonas aeruginosa* (clinical isolates from “Clínica Universidad de Navarra”, Pamplona, Spain). Cryobeads stored at −80 °C containing the previous strains were incubated in Tryptic Soy Broth (Condalab, Madrid, Spain) at 37 °C with shaking at 140 rpm for 24 h.

### 2.2. Yeast Inactivation Methods

With the aim of evaluating the postbiotic potential of yeast strains, two different inactivation methods were employed: heat and chemical treatments. For the heat treatment, strains were submitted to pasteurization-like temperatures (circa 60 °C) in a water bath or at 100 °C in steam fluent autoclave for various time points (20, 30, 40, 60 or 80 min).

For the chemical inactivation method, a novel approach previously used to inactivate bacteria [17] was applied. Briefly, yeast cells were treated with 2-bromoethylamine hydrobromide 0.1 M (BEI) (Panreac, Barcelona, Spain) and formaldehyde 0.01% (FA) at 37 °C for 24 h. However, some yeast species required a higher concentration and/or incubation time as indicated in Table 1.

Following the heat or BEI/FA treatment, 100 µL of the culture was plated into YPD agar plates and incubated at 28 °C for 5 days to confirm the total inactivation. Finally, the cells were centrifuged (6000× *g*, 20 min) and washed with ultrapure water before lyophilization and storage.

### 2.3. Bacteria–Yeast Cell Agglutination Assay

In order to assess the ability of live and inactivated yeast strains to agglutinate pathogenic bacteria, the following assay was conducted. Briefly, the yeast and bacteria strains were grown separately as previously described and diluted with sterile saline solution (0.9% salt in deionized water) to a final concentration of 8 × 10^7^ CFU/mL and 8 × 10^8^ CFU/mL, respectively. A mixture of 1 mL and 500 µL of yeast and bacterial suspension, respectively, were added to a six-well plate (Corning^®^ Costar^®^ TC-treated, Corning, NY, USA) and incubated at room temperature with no shaking. The presence or absence of agglutination was observed macroscopically.

The ability of yeast to agglutinate bacterial pathogens was also observed under a light microscope, and agglutination was determined by observing bacteria bound to the yeasts.

### 2.4. Presence of Mannoproteins

In order to investigate the potential implication of yeast mannoproteins in the agglutination of bacteria, we determined their presence by flow cytometry using fluorescent labeled-concanavalin A (ConA) using live and inactivated yeast cells. These lectins are capable of binding specifically to the α-mannopyranosyl and α–glucopyranosyl residues. For this, yeasts were grown on YPD agar, and a single colony was inoculated in YPD broth for 48 h under constant agitation conditions at 28 °C. Then, the pre-cultures were diluted in PBS (Gibco™, Waltham, MA, USA) at a concentration of 1 × 10^6^ CFU/mL. For inactivated yeasts, lyophilized material was diluted in PBS at the same concentration. All the samples were treated with BSA 2%, used as a blocking agent; stained with 10 µg/mL of ConA-AlexaFluor488 solution (Thermo Fisher Scientific, Waltham, MA, USA) for 30 min; and fixed in paraformaldehyde 4%. Samples were finally washed, resuspended in FACS buffer (PBS with FBS 2% and EDTA 2.5 mM), and analyzed by flow cytometry (Attune Cytometer, Applied Biosystem, Waltham, MA, USA), and the percentage of fluorescent staining cells and staining brightness intensity mean were assessed.

### 2.5. Mannose-Sensitive Adhesion Assay

To clarify whether the main factor in the coagglutination of bacteria was type 1 fimbriae, a Mannose-sensitive adhesion assay was performed. A similar protocol to the previous agglutination assay was performed, but bacteria were previously resuspended in a saline solution containing 2.5% D-mannose (Merck, Darmstadt, Germany), 2.5% α-L-fucose (Sigma, Madrid, Spain), or 2.5% D-galactose (Merck, Darmstadt, Germany). The effect of the different sugars in yeast coagglutination with ETEC was observed macroscopically.

### 2.6. HeLa Cell Adhesion Assay

To investigate the ability of yeasts to prevent bacterial adhesion and subsequent invasion of epithelial cells through coaggregation, a test was carried out according to the method of Boero et al. (2023) with some modifications [18]. Briefly, a non-confluent monolayer of HeLa cells was seeded at 1 × 10^5^ cells/well in 24-well plates (Corning^®^ Costar^®^ TC-treated, Corning, NY, USA) and incubated at 37 °C, 5% CO_2_ for 12 h in RPMI-1640 medium (Gibco^®^, Waltham, MA, USA) supplemented with 10% FBS (Gibco^®^, Waltham, MA, USA). Pre-cultures of bacteria strains were grown for 12 h to the stationary phase in TSB medium and diluted to reach 1 × 10^6^ CFU/mL. The yeasts were cultured for 48 h in YPD medium and adjusted to a concentration of 1 × 10^5^ CFU/mL. A bacteria–yeast (10:1) mixture was prepared in an Eppendorf tube and incubated for 30 min at room temperature. HeLa cells were then washed twice with PBS, and a volume of 450 μL of the mixture was added to each well, followed by centrifugation at 900× *g* for 10 min to facilitate the encounters of microorganisms. The plates were then incubated at 37 °C with 5% CO_2_ for 2 h. After incubation, HeLa cells were washed with PBS and lysed using a 1% Triton X-100 solution (Sigma, Spain) at room temperature. The resulting lysates were serially diluted and plated on TSA for viable bacterial colony enumeration. The results refer to yeast-free controls as 100% adhesion.

### 2.7. Statistical Analysis

Statistical analyses were performed using the computer software GraphPad Prism v.9.2.0.332 (GraphPad, San Diego, CA, USA). The significant differences were determined by ANOVA and unpaired t tests, when required.

## 3. Results

### 3.1. Yeast Inactivation

Two inactivation methods were used against stationary phase yeast cultures: a heat treatment and a non-denaturalizing method based on the use of the intercalating agent BEI/FA. The minimum conditions required for complete inactivation of each strain are shown in Table 1. Of note, *R. glutinis*, *R. mucilaginosa*, *S. cerevisiae*, *S. pombe*, and *W. anomalus* appeared to be more sensitive to heat treatment among all the studied yeasts and required lower temperature and exposure duration than *M. pulcherrima*, *P. terrestris*, *P. fermentans*, or *T. delbrueckii*. Regarding BEI/FA treatment, *S. pombe*, *T. delbrueckii*, and *W. anomalus* showed the highest sensitivity, requiring lower BEI/FA concentrations for the same exposure duration.

**Table 1:** Minimum inactivating conditions of BEI/FA and heat treatment for the different yeast strains. The table indicates the minimum concentration of BEI during 24 h treatment (M) with a constant FA concentration and the exposure duration (min) and temperature (°C) for the heat treatment necessary to achieve complete inactivation of stationary phase cultures of the indicated yeast species.

	BEI/FA	Heat Treatment	
Yeast Strain	BEI Concentration (M), 24 h	Temperature (°C)	Exposure Duration (min)
*Metschnikowia pulcherrima*	0.2	100	30
*Papiliotrema terrestris*	0.2	100	30
*Pichia fermentans*	0.2	100	30
*Rhodotorula glutinis*	0.2	60	60
*Rhodotorula mucilaginosa*	0.2	60	60
*Saccharomyces cerevisiae*	0.2	60	60
*Schizosaccharomyces pombe*	0.1	60	60
*Torulaspora delbrueckii*	0.1	100	30
*Wickerhamomyces anomalus*	0.1	60	60

### 3.2. Agglutination Assay

The capacity of live and inactivated yeasts to aggregate pathogenic bacteria was studied in vitro. Agglutination with ETEC was observed macroscopically with *P. fermentans*, *W. anomalus*, and *S. cerevisiae*, being either dead (BEI/FA or heat-treated) or alive (Supplementary Figure S1: Macroscopic and microscopic observations of coagglutination between *Saccharomyces cerevisiae* and bacterial strains). In contrast, *M. pulcherrima* and *R. mucilaginosa* were able to agglutinate ETEC only when the yeasts had been previously pasteurized or inactivated by BEI/FA, and they failed to do so when the yeasts were alive. Remarkably, *T. delbrueckii* showed agglutination exclusively after heat treatment (Table 2). Agglutination capacity was also studied against *S. flexneri,* but no yeast strain was able to agglutinate this bacterial pathogen. Live yeast strains that showed positive results with ETEC (*P. fermentans*, *W. anomalus* and *S. cerevisiae)* were also tested against *Salmonella enterica* serovar Typhimurium and *Salmonella enterica* serovar Enteritidis, and positive agglutination results with both pathogens were also observed for these yeast strains (Table 3). A non-agglutinant *Pseudomonas aeruginosa* bacteria strain was used as a negative control.

Controls w/o bacterial cells were also used to detect yeast auto-agglutination. Interestingly, auto-agglutination varied depending on whether the yeasts were alive and on the specific inactivation method employed. *S. pombe* exhibited positive auto-agglutination in both its live and inactivated forms, regardless of the inactivation method used, whereas *T. delbrueckii* showed positive auto-agglutination when alive and when inactivated using BEI/FA. These results interfered with the ability to distinguish the positive agglutination results observed with bacteria (Table 4). These findings were further confirmed by microscopic examination of Gram-stained smears (Supplementary Figure S1: Macroscopic and microscopic observations of coagglutination between *Saccharomyces cerevisiae* and bacterial strains).

### 3.3. Screening for Yeast Cell Wall Mannoprotein Content

**Figure 1:**
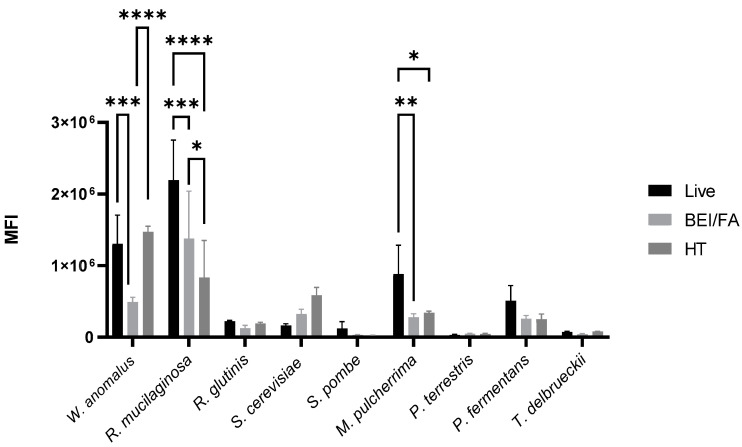
Detection of yeast mannoproteins with fluorescently labeled concanavalin A by flow cytometry. The results of mean fluorescence intensity (MFI) for each strain, live or inactivated with either chemical (BEI/FA) or heat (HT) methods, are shown (*, *p* < 0.05, **, *p* < 0.01, ***, *p* < 0.001, ****, *p* < 0.0001). Error bars represent SD (*n* = 3).

In order to characterize the agglutination mechanism and the yeast components involved in the process, the presence of mannoproteins on the different strains was screened using fluorescently labeled ConA by flow cytometry. The differences in mannoprotein content between live and inactivated yeasts are presented in Figure 1. The results showed that, among the different strains, *W. anomalus*, *R. mucilaginosa*, *M. pulcherrima*, and *P. fermentans* strains exhibited higher mannoprotein content, whereas lower mannoprotein concentrations were detected in *R. glutinis*, *S. cerevisiae*, *S. pombe*, *P. terrestris*, and *T. delbrueckii*. When comparing live and inactivated samples, live yeasts presented higher mannoprotein content compared to inactivated products, with the exception of *S. cerevisiae* and *W. anomalus,* which particularly showed lower mean fluorescence intensity when treated with the BEI/FA inactivation method.

### 3.4. Yeast-Mediated Mannose-Sensitive Agglutination of ETEC Bacteria

The implication of mannoproteins and ETEC fimbriae in agglutination was further investigated with a mannose-sensitive adhesion assay. As shown in Table 5, *S. cerevisiae* and *W. anomalus*, both live and inactivated with heat or BEI/FA, did not agglutinate with ETEC when mannose was added to the medium. In contrast, despite the presence of mannose, inactivated *R. mucilaginosa*, *M. pulcherrima*, and *T. delbrueckii* showed agglutination.

Different sugar solutions containing fucose or galactose were also used to establish the specificity to the mannose, although agglutination phenomenon was observed in all the samples, displaying a mannose mediated mechanism. Finally, inactivated *R. mucilaginosa*, *M. pulcherrima*, and *T. delbrueckii* showed auto-agglutination in mannose solution (Table 5), although this was not observed for *S. cerevisiae* or *W. anomalus*. Controls with ETEC in saline solution and yeast cells were employed.

### 3.5. Inhibition of ETEC Adhesion by Yeasts

**Figure 2:**
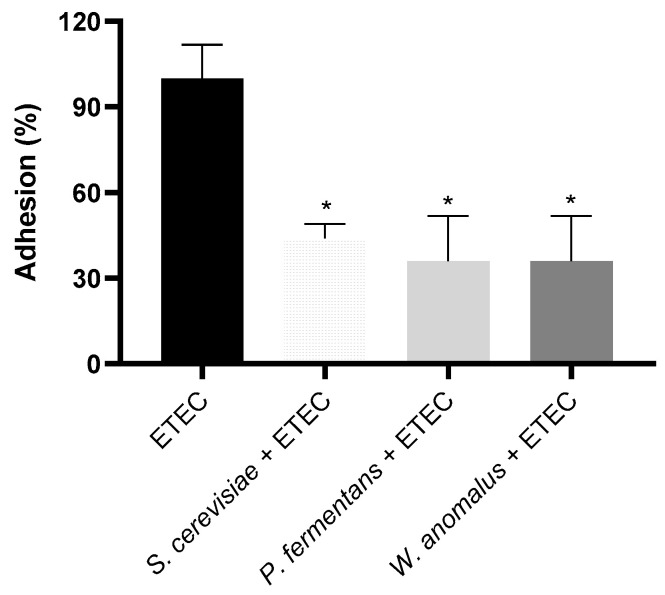
Ability of yeast strains to inhibit *Escherichia coli* ETEC adhesion to HeLa cells**.** Percentage (%) reduction in bacterial adhesion to HeLa cells compared to untreated (yeast-free) ETEC control (*, *p* < 0.05). Error bars represent SEM (*n* = 3).

In order to determine whether yeast pre-incubation was able to reduce the adherence of ETEC to HeLa cells, an adhesion assay was performed on this cell line using three different live yeasts that previously showed agglutination capacity in vitro*: S. cerevisiae*, *P. fermentans*, and *W. anomalus*. The results showed that the pre-incubation of these probiotic yeasts with ETEC significantly prevented the bacterial adhesion to HeLa cells, reducing the viable CFU by 50% (Figure 2). The results refer to yeast-free controls as 100% adhesion and show a comparison with yeast-preincubated ETEC.

## 4. Discussion

Yeast probiotics are a growing field of research, attracting interest from scientists for their potential to enhance host health. Thus, *S. cerevisiae* is among the most extensively studied yeast species and has been shown to be effective in treating various conditions, including inflammatory bowel disease and infectious diarrhea, and is the only probiotic yeast available commercially [19,20]. Based on the potential of probiotic yeasts, over recent years, investigations have been carried out with non-*Saccharomyces* species, which may provide a broader range of functionalities compared to the existing options [19]. In this context, it is important to note that while probiotics provide numerous health benefits, they also have certain limitations. These include quality inconsistencies, short shelf life, variable effects among individuals, and challenges for immunocompromised users. In fact, oral administration of probiotic preparations containing live yeast can cause severe invasive infections in immunocompromised patients, potentially leading to fungemia and subsequent sepsis as previously reported with *S. boulardii* [21]*. *Possible explanations for *S. boulardii* fungemia are translocation from oral mucous membrane into the bloodstream or from the gut to blood vessels due to digestive diseases [22]. Additionally, their application can be restricted under specific transportation and storage conditions [23]. These issues have reinforced the interest in using alternative bioactive products named as postbiotics that include inanimate microorganisms, structural parts, or secondary metabolites [9]. Postbiotics are derived from a well-defined microorganism and produced using a standardized biomass production and inactivation protocol that can be reliably reproduced. This approach enhances the opportunity to explore new species that cannot be administered live due to safety concerns [24]. Furthermore, they offer a safer alternative as they are easier to store long-term, retain stability, and allow precise control over the quantity of product administered [25]. The objective of this study was, therefore, to evaluate the probiotic and postbiotic potential of selected yeast strains in relation to their capacity to reduce the infectivity of enteric pathogens.

The most usable postbiotic, due to its ease of preparation for the industry, consists of inactivated cells. Various methods for yeast inactivation have been studied, with heat treatment with temperatures ranging from 50 °C to 100 °C being the most commonly used [26,27,28,29]. Here, we observed differences in temperature and exposure duration between the different yeast strains used in this study, *R. glutinis*, *R. mucilaginosa*, *S. cerevisiae*, *S. pomb*e, and *W. anomalus* being the most sensitive to heat treatment, requiring lower temperature and exposure duration (60 °C, 60 min). In contrast, it was necessary to use 100 °C for 30 min to inactivate *M. pulcherrima*, *P. terrestris*, *P. fermentans*, and *T. delbrueckii.* These results may be a reflection of the variations in yeast cell wall composition across species, consistent with the percentage ratios of cell wall components among yeast strains, particularly with regard to chitin, β-glucans, and mannoproteins, which are the primary surface components involved in the heat response. [30]. Furthermore, variations in heat treatment conditions could be due to a change in cell wall composition as a stress response by the yeast cells. To counteract cell wall weakening, yeasts significantly deposit chitin in the lateral walls, enhance cross-linking between chitin and β-1,3-glucan, and establish new linkages between chitin and β-1,6-glucan [31]. Additionally, heat treatment can impact the primary bioactive surface components of the cell wall, such as mannoproteins, by altering their structural conformation or causing complete denaturation [32]. Due to all these factors, in this study, as an alternative to produce whole-cell postbiotics, we employed a non-denaturing chemical inactivation method previously used by our group against bacterial species, utilizing a combination of BEI and FA [17,33]. To the best of our knowledge, this is the first report describing the efficacy of this method for yeast inactivation. Indeed, this inactivation has proven to be a safe option, as no safety concerns have arisen during its application against bacteria or viruses [17,34,35,36]. Our results demonstrated the efficacy of BEI/FA with some variations in the conditions required among the yeast species studied (Table 1). As previously mentioned for heat inactivation, the differences observed after the BEI/FA treatment could be related to the yeast cell wall composition variability. Additional research will be necessary to accurately confirm this relationship.

Due to the high morbidity and mortality of enteric pathogens and the rising issue of antibiotic resistance [15], this study explores the potential of yeast probiotics and postbiotics against major bacterial enteropathogens. Previous investigations have demonstrated the anti-infectious properties of *S. cerevisiae* against different enteric bacteria [7]. Here, we propose non-*Saccharomyces* yeast strains as preventive measures against gastrointestinal infections. Considering this, we investigated the ability of probiotic and postbiotic yeasts to agglutinate different bacterial pathogens. Firstly, we observed differences based on whether the yeast was alive and the inactivation method used. Thus, positive agglutination with ETEC was observed with both live and inactivated *W. anomalus*, *P. fermentans*, and *S. cerevisiae*. However, only the inactivated product of *R. mucilaginosa* and the heat-treated *T. delbrueckii* showed positive results. Interestingly, live yeast strains that showed agglutination with ETEC also showed agglutination with *Salmonella enterica* serovar Typhimurium and *Salmonella enterica* serovar Enteritidis. Similar results are present in the literature, where the agglutination processes between *S. cerevisiae* and enteric bacteria such as *Salmonella enterica* serovar Typhimurium or ETEC have been described [37,38]. Unfortunately, we observed the auto-agglutination phenomenon for some yeast strains, which impeded the differentiation of the coagglutination results with bacteria. This is a well-known phenomenon described for yeast as flocculation. One of the most widely accepted hypotheses for this process is the lectin-like theory, which involves the Flo1 protein. This protein is present on the outer cell wall and can interact by means of its N-terminal with mannose residues present on a neighboring yeast cell [39].

Several mechanisms of action have been proposed to explain yeast’s probiotic protection against pathogens. One possible mechanism is the interaction between yeast surface mannoproteins and type 1 fimbriae of bacteria [40]. In this study, we analyzed the presence of mannoproteins on different yeast strains using flow cytometry with fluorescently labeled concanavalin A. The results indicated that *W. anomalus*, *R. mucilaginosa*, *M. pulcherrima*, and *P. fermentans* presented higher mannoprotein content compared to *R. glutinis*, *S. cerevisiae*, *S. pombe*, *P. terrestris*, or *T. delbrueckii.* These results agree with prior investigations, where low mannoprotein concentrations for *T. delbrueckii* and *S. pombe* are described [41,42]. Moreover, we observed that mannoproteins decreased after the inactivation process. This could be attributed to the fact that heat and chemical agents can alter mannoprotein functional structure, leading to a reduction in ConA binding, as has been demonstrated after heat treatment in other studies [43].

The hypothesis regarding the involvement of type 1 fimbriae is supported by the observation that none of the yeasts, whether live or inactivated, exhibited agglutination with *S. flexneri*. While the presence of *fim* cluster, which encodes type 1 fimbriae, has been identified, it remains systematically inactive in both reference strains and clinical isolates [44]. To further confirm the role of mannoproteins and bacterial type 1 fimbriae in agglutination, we conducted a mannose-sensitive assay. The results showed that no agglutination occurred with *W. anomalus* or *S. cerevisiae* when mannose solution was used, whereas agglutination was observed with fucose or galactose solutions, demonstrating the interaction between mannoproteins and type 1 fimbriae. On the other hand, inactivated *R. mucilaginosa*, *M. pulcherrima*, and *T. delbrueckii* exhibited auto-agglutination with mannose solution, suggesting that changes in cell wall mannoproteins occur after inactivation treatment, with subsequent implications for flocculation [32].

Finally, we investigated whether this agglutination capacity is associated with a reduction in ETEC adhesion to HeLa cells and, consequently, a decrease in virulence. Our results demonstrated that a pre-incubation of *S. cerevisiae*, *W. anomalus*, or *P. fermentans* with ETEC prevented adhesion to HeLa cells, reducing CFU by 50%. This correlates with the existing literature, which reports a similar effect of yeast cells or dietary fibers containing yeast cell wall components in reducing ETEC adhesion to intestinal cells [37,45].

## 5. Conclusions

In summary, this study highlights the efficacy of yeast-based probiotic and postbiotic formulations in reducing bacterial load in vitro through the coagglutination mechanism, which effectively reduces bacterial adhesion to epithelial cells. Remarkably, we also present a novel inactivation method for the production of yeast-derived postbiotics that is safe and preserves the structural integrity of cell wall components, which is crucial for maintaining the bioactive properties of postbiotics and their functional interactions within the host. Further research is essential to comprehensively determine the postbiotic properties and their potential applications using this innovative approach.

While additional studies are required to validate these findings in vivo, the results of this investigation underscore the potential of yeast-derived products as innovative and preventive therapeutic strategies against enteric infections. This approach not only contributes to the expanding repertoire of functional biotherapeutics but also appears to be a promising tool against gastrointestinal diseases.

## Figures and Tables

**Table 2:** Agglutination ability of yeast strains, live and inactivated by heat or BEI/FA treatment, with *Escherichia coli* ETEC. The results are expressed as “−” to “+++” symbols corresponding to the agglutination levels.

Yeast Strain	Live	Inactivated	
		BEI/FA	Heat Treatment
*Wickerhamomyces anomalus*	+++	++	+++
*Torulaspora delbrueckii*	−	−	+
*Rhodotorula mucilaginosa*	−	++	++
*Pichia fermentans*	+	+++	+
*Saccharomyces cerevisiae*	+++	+++	+++
*Rhodotorula glutinis*	−	−	−
*Schizosaccharomyces pombe*	−	−	−
*Metschnikowia pulcherrima*	−	+++	+
*Papiliotrema terrestris*	−	−	−

**Table 3:** Agglutination ability of live yeast strains with *Salmonella enterica* serovar Typhimurium and *Salmonella enterica* serovar Enteritidis. The results are expressed as “−” to “+++” symbols corresponding to the agglutination levels.

Yeast Strain	*Salmonella enterica* Serovar Typhimurium	*Salmonella enterica* Serovar Enteritidis
*Wickerhamomyces anomalus*	+++	+++
*Pichia fermentans*	+++	+++
*Saccharomyces cerevisiae*	+	+

**Table 4:** Auto-agglutination results for each yeast strain, live or inactivated by heat or BEI/FA treatment. A control well with only yeast suspension was added in order to observe the ability of the different yeast strains to auto-agglutinate. The results for each of the yeast strains (live or inactivated) are expressed with “+” or “−” symbols corresponding to positive or negative auto-agglutination.

Yeast Strain	Live	Inactivated	
		BEI/FA	Heat Treatment
*Wickerhamomyces anomalus*	−	−	−
*Torulaspora delbrueckii*	+	+	−
*Rhodotorula mucilaginosa*	−	−	−
*Pichia fermentans*	−	−	−
*Saccharomyces cerevisiae*	−	−	−
*Rhodotorula glutinis*	−	+	−
*Schizosaccharomyces pombe*	+	+	+
*Metschnikowia pulcherrima*	−	−	−
*Papiliotrema terrestris*	−	−	−

**Table 5:** Agglutination and auto-agglutination results with different sugar solutions for yeast strains that showed positive results of agglutination with ETEC. The results are expressed as “−” to “+” symbols corresponding with positive or negative agglutination or auto-agglutination. The abbreviations for the sugars correspond to: MAN, mannose; FUC, fucose; GAL, galactose.

		*Saccharomyces cerevisiae*			*Wickerhamomyces anomalus*			*Rhodotorula mucilaginosa*		*Metschnikowia Pulcherrima*		*Torulaspora delbrueckii*
		Live	BEI/FA	HT	Live	BEI/FA	HT	BEI/FA	HT	BEI/FA	HT	HT
**With ETEC**	Control	+	+	+	+	+	+	+	+	+	+	+
	MAN	−	−	−	−	−	−	+	+	+	+	+
	FUC	+	+	+	+	+	+	+	+	+	+	+
	GAL	+	+	+	+	+	+	+	+	+	+	+
**Without ETEC**	Control	−	−	−	−	−	−	−	−	−	−	−
	MAN	−	−	−	−	−	−	+	+	+	+	+
	FUC	−	−	−	−	−	−	−	−	−	−	−
	GAL	−	−	−	−	−	−	−	−	−	−	−

## Data Availability

The data supporting the reported results is not available due to privacy restrictions.

## Supplementary Materials

The following supporting information can be downloaded at: https://www.mdpi.com/article/10.3390/pathogens14020113/s1, Figure S1: Macroscopic and microscopic observations of coagglutination between *Saccharomyces cerevisiae* and bacterial strains.
