# Supplementary material for: Yeast Strains as Probiotic and Postbiotic Agents for the Agglutination of Enteric Pathogens: A Preventive Approach
Source: Pathogens. 2025 Jan 24;14(2):113. doi: 10.3390/pathogens14020113 (PMC11858296; doi:10.3390/pathogens14020113)
Supplement: Supplementary file 1 [file pathogens-14-00113-s001.zip › pathogens-3411012-supplementary.pdf]

Supplementary material.

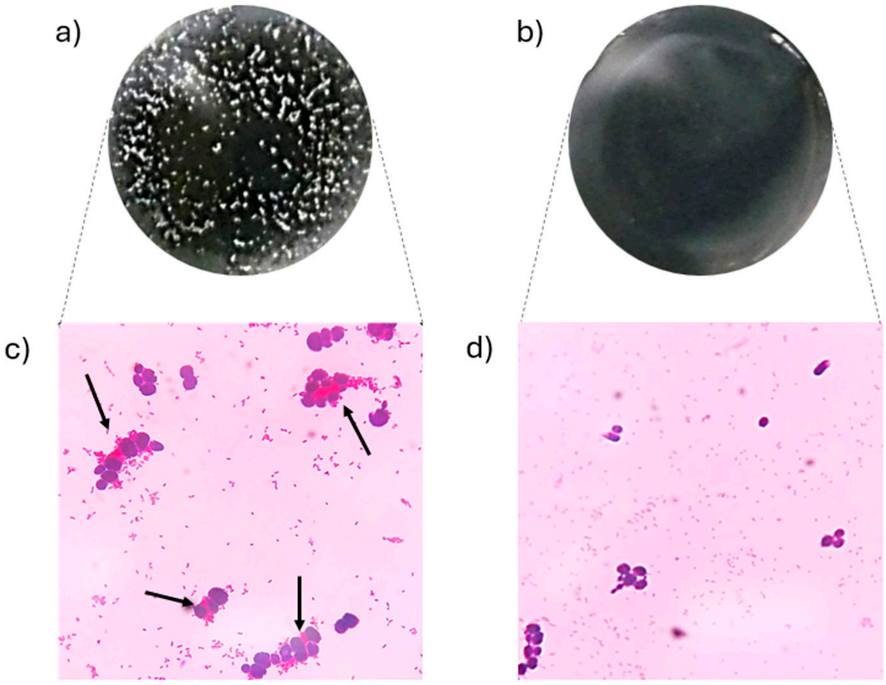

Figure S1. **Macroscopic and microscopic observations of coagglutination between *Saccharomyces cerevisiae* and bacterial strains.** a) Macroscopic evidence of coagglutination between *S. cerevisiae* and Enterotoxigenic *Escherichia coli*. b) Absence of coagglutination between *S. cerevisiae* and *Pseudomonas aeruginosa*. c) Microscopic confirmation of coagglutination observed in a), with arrows indicating clusters of *S. cerevisiae* and ETEC. d) Microscopic observation of the lack of co-agglutination in b), showing no interaction between *S. cerevisiae* and *P. aeruginosa*.
